# Financial recommendations on Reddit, stock returns and cumulative prospect theory

**DOI:** 10.1007/s42521-023-00084-y

**Published:** 2023-04-18

**Authors:** Felix Reichenbach, Martin Walther

**Affiliations:** Chair of Finance and Investment, Technische Universität Berlin, Sec. H 64, Straße des 17. Juni 135, 10623 Berlin, Germany

**Keywords:** Behavioral finance, Prospect theory, Reddit, Social media, Stock return, G11, G12, G14, G41

## Abstract

This study investigates stock recommendations from the three largest finance subreddits on Reddit: wallstreetbets, investing and stocks. A simple strategy that buys recommended stocks weighted by the number of posts per day yields a portfolio with higher average returns at the expense of higher risks than the market for all holding periods, i.e., unfavorable Sharpe ratios. Furthermore, the strategy leads to positive (insignificant) short-term and negative (significant) long-term alphas when considering common risk factors. This is consistent with the idea of “meme stocks”, meaning that the recommended stocks are artificially inflated in the short term when they are recommended, and that the posts contain no information about long-term success. However, it is likely that Reddit users, especially on the subreddit wallstreetbets, have preferences for bets which are not captured by the mean–variance framework. Therefore, we draw on cumulative prospect theory (CPT). We find that the CPT-valuations of the Reddit portfolio exceed those of the market, which may explain the persistent attractiveness for investors to follow social media stock recommendations despite the unfavorable risk-return ratio.

## Introduction

Social media platforms, such as Reddit, offer a place to share and discuss ideas about investment strategies. Recently the subreddit *wallstreetbets* received considerable attention due to the short squeeze of GameStop in January 2021 and triggered a discussion about whether Reddit posts are only memes or contain valuable information. In this study, we examine whether it is reasonable to follow the recommendations on the three largest subreddits that deal with investment strategies, i.e., *wallstreetbets* (WSB), *stocks*, and *investing*. We use a comprehensive data set of submissions to these subreddits and implement a simple strategy that buys recommended stocks, with the portfolio weights proportional to the number of posts. We then study the resulting return distribution through the lens of both modern portfolio theory and prospect theory to investigate whether following stock recommendations from Reddit would yield portfolios with desirable characteristics. The consideration of Reddit is particularly relevant due to its recent significant impact on financial markets, and the controversy surrounding the behavior of Redditors in the media and in research:

In the financial press, the WSB-related short squeeze of GameStop was often referred to as “GameStop mania” or “a bubble”,^1^ echoing the idea of the “madness of crowds” (in the spirit of Mackay, 1841) and thus suggesting that following stock recommendations on Reddit may not be advisable. However, moderators of WSB argue that their culture is misunderstood and that “retail investors can be every bit as sophisticated as institutional investors” because their community consists of “researchers, mathematicians, momentum traders, gamblers, and so much more” (Reddit, 2021) invoking the idea of the wisdom of crowds (see Surowiecki, 2004).

Two main narratives can be identified in academic research as well: First, many authors argue that retail investors exhibit herd behavior (Lyócsa et al., 2022; Semenova & Winkler, 2021) and that social media posts either do not add value (Chacon et al., 2022) or even have a negative impact on market stability (Corbet et al., 2021). Conversely, some recent evidence suggests, that social media investors are well-informed and skilled and realized above-average returns (Bradley et al., 2021; Buz & Melo, 2021; Hasso et al., 2021; Welch, 2022). Therefore, in this study, we want to further investigate whether it is reasonable to follow recommendations on social media.

The methodology of our study is as follows. We extract Reddit posts that contain a ticker of a stock. Each trading day, recommended stocks are bought and weighted with the number of posts. This means that stocks that are discussed more frequently constitute a larger percentage of the portfolio. We then calculate the returns of the “Reddit portfolio” for different time horizons. In the first step, we consider the mean and variance of the portfolio return. While the average return of the Reddit portfolio exceeds the market portfolio return, the variance is much higher. This suggests that the implemented strategy without additional diversification is not worthwhile when considering the mean–variance framework. Furthermore, when calculating excess returns in a model that incorporates common risk factors, alphas are insignificant for short holding periods, followed by significant negative long-term excess returns.

However, recent literature indicates that some investors have preferences for lottery-like stocks (e.g., Eraker & Ready, 2015). The returns of the Reddit portfolio are highly skewed, meaning that they may be interesting for such investors. We argue that members of communities such as *wallstreetbets* are likely to have preferences for betting, which are not captured by the mean–variance framework. Therefore, we make use of Tversky and Kahneman’s (1992) cumulative prospect theory (CPT) and calculate the valuations according to CPT for the Reddit portfolio. We find that the Reddit portfolio yields higher CPT values than the market, which indicates that following financial recommendations on Reddit may be reasonable for investors with preferences for lottery-like stocks as in CPT.

This result has two important implications. First, following recommendations on Reddit’s financial communities may be reasonable for investors with CPT preferences. This suggests that the recommendations contain information. Second, WSB features more than 13 million members, which means that a relevant mass of investors is likely to have preferences that are better captured by CPT than the mean–variance framework. This finding may be important when building models to describe and analyze the behavior of individuals and markets.

The remainder of this study is organized as follows: Sect. 2 reviews the literature on social media and financial markets as well as CPT and then explains the research objective. Section 3 describes the dataset and outlines the methods used. Section 4 presents the results. Section 5 discusses the implications and limitations. Section 6 concludes.

## Theoretical background and hypotheses

In this section, we briefly review the current literature on social media and its impact on financial markets. Furthermore, we explain CPT and present our research question.

### Social media and stock returns

The informativeness of social media posts is discussed in numerous studies with differing results.

Several studies provide evidence that social media platforms can efficiently aggregate individual wisdom, and therefore be valuable in making informed decisions. We will refer to this idea as the wisdom of crowds (Surowiecki, 2004). In the early 2000s, Antweiler and Frank (2004) showed that internet posts have a significant but small effect on stock returns and can help predict volatility. Welch (2022) analyzes the “wisdom of the Robinhood crowd” and finds that an aggregate portfolio of all stocks held by users of the broker app (which is often used by WSB members) generates alpha. Chen et al. (2014) use data from the investment platform Seeking Alpha and find that shared opinions predict future stock returns and earnings surprises. This result is robust to controlling for the effects of traditional sources of financial advice. Relatedly, Farrell et al. (2022) make use of the editorial delay between report submission and publication on Seeking Alpha to show that social media can contribute to better inform retail investors. Bartov et al. (2018) investigate a sample of tweets from 2009 to 2012. They find that the aggregate opinion on Twitter predicts future quarterly earnings and announcement returns. Tang (2018) shows that product information by customers on Twitter can predict upcoming sales and unexpected sales growth at the firm level.

In contrast, Giannini et al. (2018) find a negative relationship between the sentiment of Twitter posts and future returns, using tweets from November 2008 to 2011. They conclude that simply relying on social media sentiment is dangerous and likely to be an unprofitable trading strategy. Jia et al. (2020) also examine Twitter data. They find that merger rumors that are accompanied by greater Twitter activity yield stronger price reactions although the number of tweets does not predict the probability of merger realization. These studies indicate that social media can also have negative effects on decisions.

The finance-related subreddits on Reddit, especially WSB, are also discussed in recent studies. Boylston et al. (2021) examine how WSB has become one of the largest communities on Reddit with a very loyal user base. Agrawal et al. (2022) analyze the thematic and linguistic characteristics of the three subreddits that we consider. They find that WSB mainly discusses high-risk investments, while *stocks* discusses conventional strategies and *investing* focuses on low-risk long-term investments. When investigating the usefulness of Reddit posts, several studies focus on short squeezes. Betzer and Harries (2022) investigate the relationship between Reddit posts and GameStop retail trading. They find a significant increase in trading activity after Reddit posts but no significant relationship between Reddit posts and realized abnormal returns. They conclude that the posts are not informative. Lyócsa et al. (2022) consider four short squeezes (GameStop, AMC Entertainment Holdings, Blackberry and Nokia). They find that the activity on WSB relative to Google searches can explain part of the next day’s price variation. Costola et al. (2021) develop a method to identify “meme stocks” that are currently discussed in social media. They call this phase, in which stock prices are driven by momentum originating from the herding of social media users, “mementum”. In contrast to these studies, we do not focus on a few but a large number of stocks. Furthermore, we do not investigate whether posts can be used to predict the price movements of individual stocks but consider the return distribution of a simple investment strategy.

Related approaches have been used in recent studies. Agrawal et al. (2022) examine WSB posts between January 2019 and April 2021. They find that following proactive buy signals would have achieved better results than randomly or equally distributing investments, both long and short-term. Bradley et al. (2021) focus on due diligence reports on WSB from July 2018 to June 2021. They find that the reports can predict one-month ahead returns, earnings forecast revisions, and earnings surprises until 2020. After the GameStop short squeeze in 2021, the reports lose their predictive power. In contrast to these two studies, we consider a much longer time horizon from 2008 to 2022, and more than one subreddit. Furthermore, we consider the entire return distribution and value it according to CPT.

Methodically, our approach is similar to the one of Chacon et al. (2022). They consider posts on WSB from 2012 to the first quarter of 2021, in which the GameStop short squeeze took place. They find a positive relation between posts and abnormal trading volume, suggesting that Reddit posts may induce trading activity. Furthermore, a strategy that goes long buy recommendation and short sell recommendations each day is considered. Buy and sell recommendations are classified by using a simple word list. In the Fama–French five-factor plus momentum model, the portfolio does not generate positive alphas. This implies that investing in the Reddit-based portfolio is not profitable from a risk-adjusted perspective and suggests that following the recommendations on WSB would not be reasonable.

However, even if we assume that following this advice is irrational in a mean–variance framework it is likely that the primary goal of the members of WSB, as the name *wallstreetbets* suggests, is to find attractive targets for betting. Therefore, insignificant alphas do not necessarily imply that investors have not achieved their goals. Several studies have documented a preference of investors for skewness, which is not captured by the mean–variance framework. We will shortly discuss these at the end of Sect. 2.2.

The preferences for skewness can be linked to the probability weighting in CPT, which we will explain in the next section. In this context, Barberis et al. (2016) calculate the CPT values of stocks based on past returns. They find that these values are negatively related to subsequent returns. This implies that some investors increase their holdings of stocks with high CPT values and decrease their holdings of stocks with low CPT values, meaning that they have preferences as modeled by CPT. These results suggest that investors deliberately pick stocks that have negative alphas because they have a preference for their lottery-like characteristics. Therefore, CPT might explain why the recommendations on Reddit are reasonable and contain information, although they do not generate significant alphas according to Chacon et al. (2022).

### Cumulative prospect theory

In this subsection, we introduce the cumulative prospect theory (CPT) proposed by Tversky and Kahneman (1992).

We use a similar notation to Barberis et al. (2016). First, the returns $x$ are sorted in ascending order. $m$ and $n$ denote the number of negative and positive returns, respectively. Negative returns are denoted by negative indices, which means that ${x}_{-m}$ is the smallest return. Positive returns are denoted by positive indices, meaning that ${x}_{n}$ is the highest return. ${p}_{i}$ stands for the probability of the respective return. In CPT, investors assign the following value to a gamble:

1 $$\sum_{i=-m}^{n}{\pi }_{i}v\left({x}_{i}\right)$$

The so-called value function is defined as follows:

2 $$v\left(x\right)=\left\{\begin{array}{c}{x}^{\alpha }, \,for \,x\ge 0\\ -\lambda {\left(-x\right)}^{\beta }, \,for \,x<0\end{array}\right.$$

**Fig. 1:**
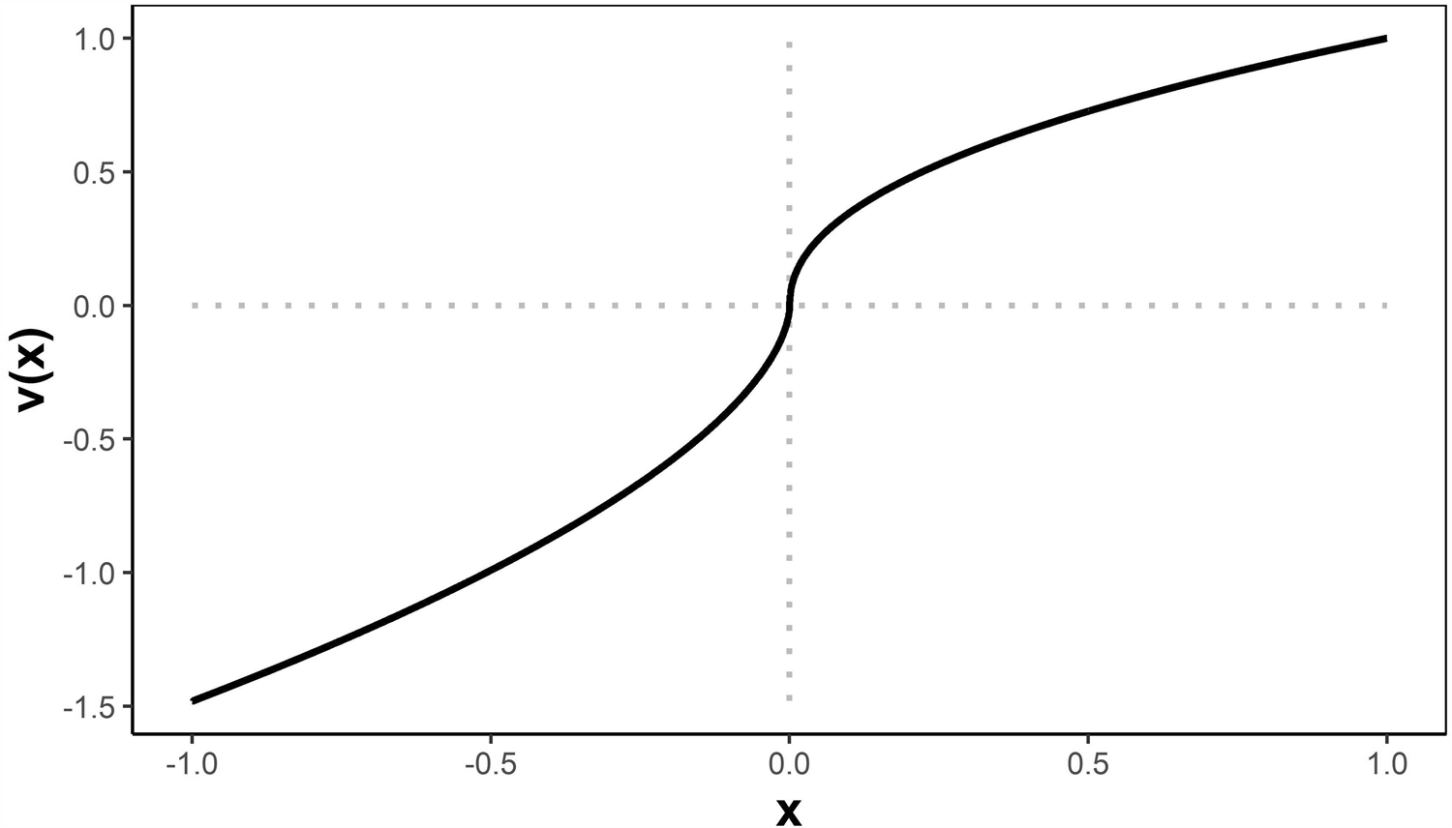
Value function using the average worldwide estimates of Rieger et al. (2017): $\alpha =0.46,\beta =0.58$ and $\lambda =1.48.$

Figures 1 depicts a value function using the average worldwide estimates of Rieger et al. (2017).

In contrast to the expected utility framework, CPT does not use final wealth but gains and losses relative to a reference point. In our study, we use the return of the market portfolio as a reference. This means that returns that are higher than the corresponding returns of the market portfolio are considered a gain and negative returns in excess of the market are classified as a loss. The value function has a kink at the origin because investors are more sensitive to losses ($\lambda >1$), which is often referred to as loss aversion. Furthermore, the value function is convex for losses and concave for gains, implying that investors are risk averse in gains and risk seeking in losses. This effect is often referred to as the reflection effect.

The probability weights ${\pi }_{i}$ are calculated using weighting functions ${w}^{\pm }(P)$:

3 $${\pi }_{i}=\left\{\begin{array}{c}{w}^{+}\left({p}_{i}+...+{p}_{n}\right)-{w}^{+}\left({p}_{i+1}+...+{p}_{n}\right), \,for \,i\ge 0\\ {w}^{-}\left({p}_{-m}+...+{p}_{i}\right)-{w}^{-}\left({p}_{-m}+...+{p}_{i-1}\right), \,for \,i<0\end{array}\right.$$

with

4 $${w}^{+}\left(P\right)=\frac{{P}^{\gamma }}{ {\left({P}^{\gamma }+{\left(1-P\right)}^{\gamma }\right)}^{1/\gamma } } \,{\text{and}} \,{w}^{-}\left(P\right)=\frac{{P}^{\delta }}{ {\left({P}^{\delta }+{\left(1-P\right)}^{\delta }\right)}^{1/\delta }}$$

**Fig. 2:**
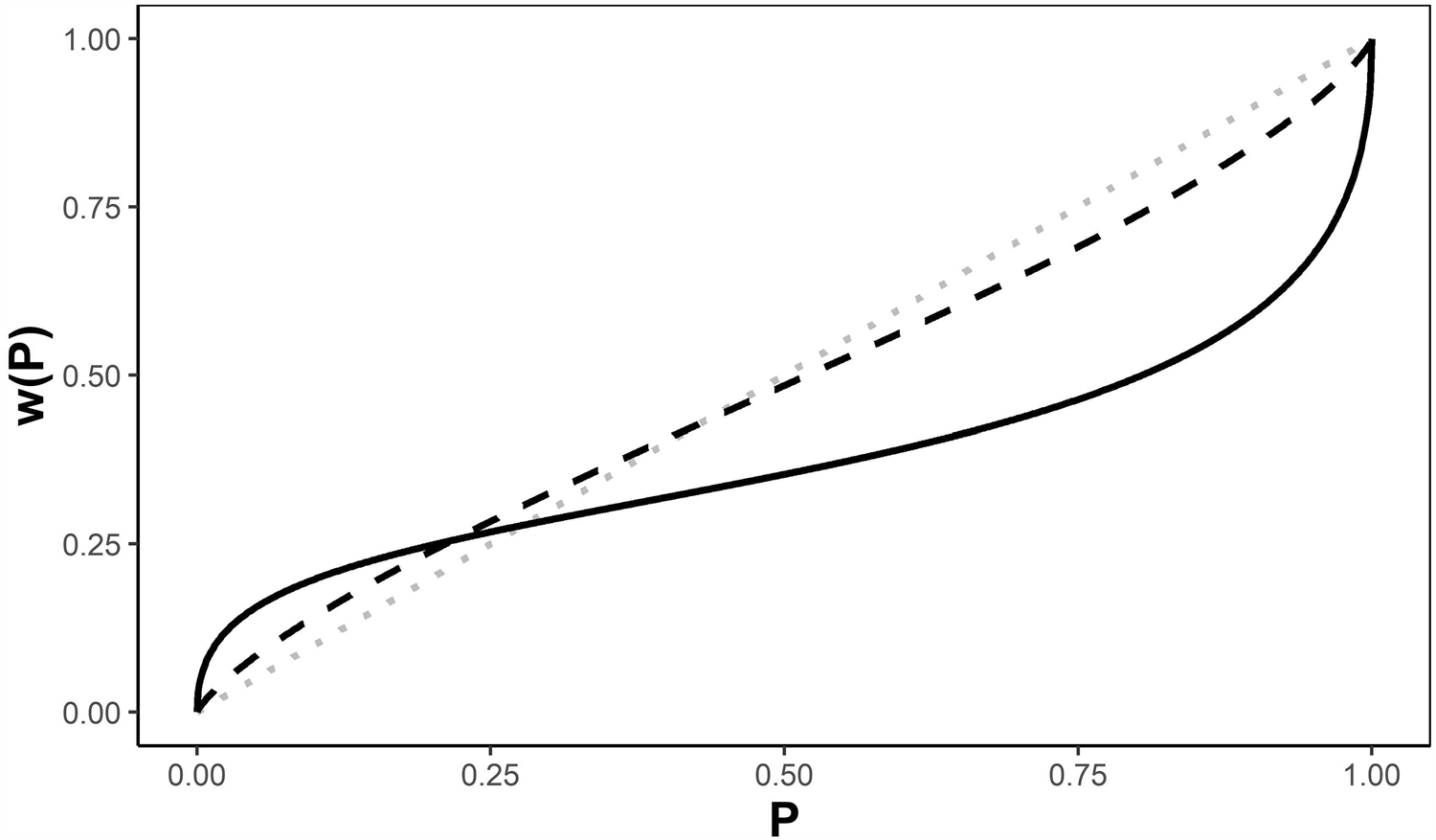
Weighting functions of the CPT using the worldwide estimates of Rieger et al. (2017): $\gamma =0.50$ (solid black line) and $\delta =0.81$ (dashed black line). Objective probability weighing is depicted as the dotted grey line

Figure 2 illustrates one of the used weighting functions. In CPT, investors do not use objective probabilities (dotted grey line) but transformed probabilities via the weighting functions. The probability weights for gains (${x}_{i}>0$) are calculated as follows. First, the total probabilities for all positive returns equal to or higher than ${x}_{i}$ are computed. Then, the total probabilities for all returns are strictly higher than ${x}_{i}$ are calculated. Next, the weighting function ${w}^{+}( \cdot )$ is applied to each of these probabilities. Finally, the difference between these two values is the probability weight. Analogously, the probability weight for losses is calculated as the difference between the weighted total probability that the return is equal or lower than ${x}_{i}$ and the weighted total probability that the return is strictly lower than ${x}_{i}$. As a result of this probability weighting, investors overweight the tails of the distributions. Thus, CPT can explain why investors simultaneously demand lotteries and insurances. Furthermore, the CPT values of a portfolio are higher when the returns are positively skewed. Overall, the CPT-values of a portfolio are likely to be high when the expected returns are high, the standard deviations are small (loss aversion), and skewness is high (probability weighting). A stock’s returns are highly positively skewed when small losses occur frequently while there are a few large gains. The overweighting of these few large gains in CPT captures the intuition that the members of subreddits like *wallstreetbets* may have preferences for betting. Therefore, a valuation according to CPT of the return distribution of a portfolio built with Reddit data is worth investigating.

CPT has been linked to investor behavior and asset prices in several studies. Barberis and Huang (2008) focus on probability weighting and find that, under CPT, a stock’s own skewness can be priced. Specifically, positively skewed assets can earn a negative average excess return and still be bought by investors. This corresponds to our idea that Redditors might recommend stocks with unfavorable alphas because they have the potential for very high returns, i.e., are positively skewed. Preferences for skewness are documented in a number of studies (e.g., Mitton & Vorkink, 2007; Kumar, 2009; Amaya et al., 2015). Bali et al. (2011) find a statistically and economically significant relationship between extremely positive returns, i.e., the maximum daily return of the prior month, and future returns. Their results are consistent with preferences for lottery-like payoffs causing under-diversification. Fong and Toh (2014) confirm the “MAX effect” of Bali et al. (2011) using recent data. Furthermore, they show that the MAX effect is mainly driven by the poor performance of high MAX stocks, and past investor sentiment. Eraker and Ready, (2015) show that over-the-counter (OTC) stock returns are highly positively skewed and negative on average, which can be explained by the model of Barberis and Huang (2008). Barberis et al. (2016) show that stocks with high CPT valuations of past returns yield lower subsequent returns.

Furthermore, recent studies provide support for reference-dependence as in prospect theory, i.e., risk-seeking behavior when facing prior losses, and risk aversion when facing prior gains. Wang et al. (2017) find that among firms with prior capital gains, there is a weak positive relation between risk and return. In contrast, stocks of firms with capital losses feature an inverted risk-return relation. Relatedly, An et al. (2020) find that anomalies related to lottery features are strong when investors have lost money, while they are reversed after gains. Walther and Münster (2021) show that beta given a gain yields positive conditional risk premiums while beta given a loss yields negative conditional risk premiums.

He and Zhou (2011) built a portfolio selection model that incorporates all key features of CPT, i.e., a reference point, an S-shaped value function, and probability weighting. Barberis et al. (2021) also consider all relevant elements of CPT. Their model predicts alphas that are consistent with the majority of 23 prominent anomalies, which indicates that CPT is a promising framework to study investor behavior. Recently, Bali et al. (2021) show that high investor attention and intense social interaction amplify the lottery anomaly among stocks dominated by retail investors. They conclude that attention and social interaction contribute to investors’ attraction to lottery-like stocks. Our study relates attention and social interactions on Reddit to subsequent CPT values and can therefore also contribute to this stream of literature.

### Research questions and contributions

The main aim of this study is to examine whether it is reasonable to use buy recommendations on the three largest finance subreddits on Reddit. We therefore analyze the distribution of returns resulting from following these recommendations to determine if such portfolios possess desirable characteristics both in the mean–variance frameworks and CPT.

Desirable characteristics in terms of the mean–variance framework are a high average return combined with low risk (i.e., volatility). Therefore, we compare these measures for the recommended stocks and the market portfolio to examine whether it would have been worthwhile for the average investor to follow the stock recommendations on Reddit. In the second step, we also consider common risk factors and determine excess returns.

However, even if using financial advice from Reddit is not worthwhile from a risk-return standpoint, it could still be attractive for investors with preferences as in CPT. Therefore, we analyze the extremes and skewness of the distribution to account for the potential benefit that investors interested in lottery-like stocks might obtain. Then, we calculate the CPT values of the realized post-recommendation returns in excess of the market of each portfolio for different holding periods and parameters. A positive CPT value is favorable because it is higher than the CPT value of the market portfolio (which always has a CPT value of 0 because we consider returns in excess of the market) and means that an investor would prefer to invest in the Reddit portfolio. Note that we use a portfolio’s future, rather than past returns as of the date of the submission to calculate CPT valuations. In summary, we backtest a strategy using buy recommendations on Reddit identified by sentiment analysis for investors with preferences according to the mean–variance framework or CPT, respectively.^2^

Furthermore, when considering different holding periods, the theoretical background yields the following additional research questions. First, regarding the informativeness of Reddit posts in terms of the mean–variance framework, we consider the two opposing positions derived from the literature (see Sect. 2.1.): If Redditors as a group can identify mispriced opportunities in the stock market through community discussion (wisdom of the crowds), we should observe significant outperformance over all holding periods for buy recommendations. The opposite position is that the prices are artificially inflated by the herding of subreddit members (who push so-called “meme stocks”) until they lose interest, and the stock price returns to its fundamental value in the long-term. This pattern would imply that the madness of crowds is more appropriate to describe the behavior of Redditors. Therefore, we would expect that in this case, abnormal excess returns of the recommended stocks can be observed in the short run. However, these would disappear or even become negative in the long run once the stock is no longer popular.

Second, we analyze whether the different characteristics of the three subreddits as described by Agrawal et al. (2022) are reflected in the return distribution of the resulting portfolios. One would expect that WSB users are more focused on short-term high-risk investments while *investing* users prefer long-term outperformance.

The contribution to the literature and the distinction between this study and existing research, especially Chacon et al. (2022), is fourfold: First, we compute average returns, variance, and excess returns over the entire period across three different subreddits and different holding periods as opposed to individual stocks or subreddits. Second, we consider the distribution of returns in more detail to investigate their lottery properties. Third, we improve the recognition of buy recommendations using sentiment analysis. Fourth, we examine the return distribution using CPT to explore whether investors would follow Reddit posts even if the risk-return ratio is unfavorable.

## Data and methodology

In this section, we present the data set and explain our methodology. First, we briefly introduce the Reddit platform and discuss our data sources. Next, we describe the identification of buy recommendations via sentiment analysis and present descriptive statistics of the resulting dataset. We then outline our method for the portfolio formation, the calculation of the risk-adjusted performance measure (alpha), and the calculation of CPT valuations.

### Reddit and stock return data

Reddit was founded in 2005 as a social news aggregation platform, with the idea of representing the “frontpage of the internet” where users can find and share content from external sources. However, over the years, Reddit increasingly transformed into a social media platform as the focus moved to user-generated content (Singer et al., 2014). The platform gives its users the opportunity to organize in subreddits where they can create their own communities with a distinct culture and a set of rules that is enforced by moderators. In these subreddits, users (called Redditors) can submit posts (submissions) with which the other users can interact via comments, up- and downvoting or by giving awards. The most popular posts will be displayed first on the subreddit's page until they drop in rank as they become older. Users can subscribe to subreddits to become members of the communities and to be able to submit their own content.The largest subreddits dedicated to investing or trading, as of January 2023, are r/wallstreetbets (13.5 million subscribers), r/stocks (5.1 million), and r/investing (2.1 million). *Investing* is the oldest subreddit (created in March 2008) and focuses on long-term strategies and portfolio allocation decisions. It is followed by *stocks* (June 2008), which mainly discusses individual stocks. WSB was created in January 2012 as an alternative to *investing* and was originally planned to be specifically for gamblers and speculators opposed to long-term investors.^3^ While it remained a relatively small subreddit throughout the first years, the number of submissions and subscribers steadily grew. In January 2021, induced by the GME short squeeze, WSB gained more than six million subscribers.

WSB describes itself as a community “like 4chan found a Bloomberg terminal” and features a distinct culture including its own language and memes (Chohan, 2021). Users often call themself “retards”, “degenerates” or “apes” and regularly declare to “just like the stock” instead of basing their trading decisions on fundamental research, which seems to confirm the pre-conception that they are unsophisticated gamblers.

We use the Pushshift Reddit API (Baumgartner et al., 2020) to gather all available submissions from the three subreddits. Pushshift archives all submissions and comments in regular time intervals and makes it available to researchers free of charge. The data set includes historical data going back to the inception of Reddit in 2005 and is frequently used in academic research (e.g., Buz & Melo, 2021; Lyócsa et al., 2022). We only use submissions in our analysis that were not immediately (automatically) deleted because they violate community rules. The period under consideration in this study extends from the first post in 2008 to August 31, 2022.

As a source for financial data, Refinitive Eikon is used. Since most users are US-based and nearly all discussed stocks are domestic, the analysis is limited to stocks listed on a US stock exchange. We filter for all active and historical securities in the category *equities* listed on the NYSE and NASDAQ as their primary listing and obtain their daily total return index. In addition, unadjusted historical stock prices from the Eikon database are used to identify the periods when the share prices in question fell below $1. Such penny stocks should not be discussed under WSB rules (Reddit, 2021) and usually have very low market capitalization and liquidity. We therefore exclude these stocks in the respective periods. The same applies to microcap stocks with a market capitalization below $10 million on the day of the posting. Finally, daily factor returns from Kenneth French’s website are used to take the factor exposure of the portfolios into account (French, 2022).

### Identifying stock tickers and buy recommendations

The Reddit data set does not provide data on buy or sell recommendations of specific stocks. Hence, the stocks mentioned in a post have to be identified, to link them to the subsequent stock performance. A review of the posts shows that the overwhelming majority of authors use the symbols (also called stock tickers) of the discussed securities instead of their names (e.g., TSLA instead of Tesla). Therefore, the titles of the submissions are searched for stock tickers. The self text field is not used because it often contains other symbols outside the primary subject of the post. Since it is not uncommon on Reddit to capitalize words completely to emphasize them, and many English words are also used as stock tickers (e.g., BOOM is the symbol of DMC Global), we check all potential tickers against a dictionary containing more than 120,000 English words.^4^ Symbols with a possible double meaning are only considered if they are preceded by a $ sign. The same applies to all single-letter symbols (such as $F for Ford) and common abbreviations that are not included in the dictionary (e.g., DD for Due Diligence and DuPont de Nemours). Subsequently, we cross-reference the extracted symbols from the WSB submission titles with the total return index from Eikon and calculate returns for different time periods.

To identify buy recommendations, we use VADER sentiment analysis in Python (Hutto & Gilbert, 2014). It is a tool that calculates a sentiment score of a text based on a lexicon that is specifically designed for social media. The lexicon contains a list of words and emojis that are rated on a scale from – 4 to + 4. A valence score of – 4 represents the most negative and + 4 the most positive words, respectively. Sentiment scores are computed by summing the scores of the words in a text and then normalizing them to a value between – 1 and + 1, where + 1 represents a very positive text, 0 represents a neutral text and – 1 represents a very negative text.

Research on textual analysis has shown that words often have different meaning when used in a financial context. Therefore, we update the lexicon with the words from Loughran and McDonald’s (2011) positive and negative word lists. Furthermore, we consider the 1000 most frequently used words in the titles and posts of our sample and add relevant words (and emojis)^5^ to the lexicon. Valence scores are assigned manually. We then test different combinations of these word lists. Furthermore, we optimize the sentiment score threshold that is required for a post to be considered a buy (or sell) signal.

To quantify the performance of our classification and to optimize hyperparameters, we manually classify 8000 posts that contain a ticker into buy signals, sell signals, or no signals. Of these, 302 posts were either unclear in content or did not contain a valid ticker upon closer inspection, resulting in a total of 7698 posts being evaluated. Posts were considered buy signals if they contained either (1) direct buy recommendations, (2) a statement that the author or another person holds or has recently bought shares, (3) positive news about the company or the stock, (4) a statement that the share price is currently rising or will rise in the future or similar statements, and vice versa for sell signals. We obtain 3647 buy-signals and 596 sell-signals, which correspond to roughly 47% buy signals or 8% sell signals.

We optimize hyperparameters by maximizing the F_0.5_ score (Sasaki, 2007):

5 $${F}_{0.5}=\frac{{(1+0.5}^{2})\cdot precision\cdot recall}{{0.5}^{2}\cdot precision+recall}$$

This measure considers precision, that is the fraction of true buy-signals among the posts classified as buy signals, as twice as important as recall, which is the fraction of true buy signals that are successfully classified as buy signals. For our analysis, recall is less important than precision as our sample contains a large number of posts. Furthermore, misclassifying sell signals as buy signals or vice versa is more costly than misclassifying posts that do not contain a clear signal (“signalless”) because sell and buy signals are likely to have opposite effects, which can substantially dilute our results. Therefore, we use the mean of the overall precision and the precision without the consideration of signalless posts, that is the fraction of correctly classified buy signals among true buy and sell signals, as precision in Eq. 5. To sum up, our measure places more weight on not misclassifying than on finding all buy or sell signals. Furthermore, misclassifications of sell signals as buy signals and vice versa are weighted more heavily than misclassifications of neutral posts.

The F_0.5_ score shows that using a wordlist that is updated with the words from Loughran and McDonald (2011) and our manual lists perform best. The optimal thresholds are 0.35 for buy signals and − 0.6 for sell signals. This means that posts with a sentiment score of above 0.35 are considered buy signals while all posts with a score of less than − 0.6 are classified as sell signals. In the following paragraph, we discuss the performance of our approach when compared to the approach of Chacon et al. (2022) who use short custom word lists to identify sell and buy signals.^6^

Our approach leads to 2206 correct buy signal classifications, which is a recall of more than 60%. The recall when using the approach of Chacon et al. (2022) is 28.57%, implying that our approach is able to detect significantly more buy signals. Furthermore, our approach leads to 1013 misclassified buys, which implies a precision of 68.53% (compared to 62.70% for Chacon et al., 2022). Of these 1013 misclassifications, only 113 are truly sell signals. This means that the precision without the consideration of signalless posts amounts to 95.13% (92.38%). Thus, regarding buy signals, our approach results in a significantly higher recall while also having slightly higher precisions.

As only a few posts can be considered sell signals, both our and Chacon et al. (2022) approach perform very poorly in identifying sell signals. Our approach detects 212 of the true sell signals, which is a recall of 35.57%, and classifies a total of 767 posts as sell signals, meaning that the precision is only 27.64%. The precision of the Chacon et al. (2022) approach is similar and amounts to 27.27%. However, the recall is even worse and amounts to less than 10%. Therefore, a separate analysis of sell signals identified with the help of word lists is likely to be not meaningful.

When applied to the entire data set of 313,896 identified stock recommendations, our approach leads to 152,673 classifications as a buy signal. This represents 48.64% of all recommendations. Given the precisions in our sample, these posts should contain more than 100,000 true buy signals and less than 5400 true sell signals that are misclassified as buy signals. Regarding sell signals, 33,420 posts are classified as sell signals, which corresponds to 10.65% of all posts. Due to the poor performance of the classification approach, it is likely that only around 9000 of these are true sell signals. However, our analysis focuses on buy recommendations, as these make up the overwhelming majority of posts and can be correctly identified with a high degree of certainty.

### Descriptive statistics

Table 1 presents the descriptive statistics of the resulting Reddit data set, including the number of buy recommendations made in each subreddit each year, and the number of subscribers at the time of the first buy recommendation in each year. Only posts that can be clearly assigned to a specific stock ticker, do not refer to penny stocks or microcaps, have not been deleted automatically, and have been posted at most 24 h before market close are considered. Buy recommendations are determined using sentiment analysis, as explained in the previous subsection.

**Table 1:** Descriptive statistics of the Reddit data set

Year	Number of buy recommendations			Number of subreddit subscribers		
	Investing	Stocks	Wallstreetbets	Investing	Stocks	Wallstreetbets
Pre-2015	1015	549	477	–	–	–
2015	654	774	1033	–	–	–
2016	689	1042	2424	–	–	–
2017	627	1366	4031	–	–	–
2018	554	1147	6703	499,740	225,180	236,979
2019	458	822	4421	644,372	307,657	455,048
2020	1235	4004	17,278	859,611	459,168	772,632
2021	727	3104	88,135	1,253,712	1,002,968	1,757,260
2022	107	870	8427	1,974,999	3,460,244	11,415,586
Sum	6066	13,678	132,929	–	–	–

The left panel displays the number of buy recommendations per year that were made in the respective subreddit. For this purpose, only posts that could be clearly assigned to the mentioned ticker of a stock, that did not deal with penny stocks or microcaps and have not been deleted by moderators or bots are taken into account. In addition, we only consider posts on stocks that can be traded at the closing price in the next 24 h. Buy recommendations are determined using sentiment analysis (as described in Sect. 3.2). The panel on the right contains the number of subscribers at the time of the first buy recommendation in the given year when data is available. Note that our data set only includes posts up to August 31, 2022

According to our data, the WSB subreddit had the highest number of buy recommendations in each year since 2015, with a total of 132,929. The investing subreddit had 6066 buy signals, while the stocks subreddit had 13,678. In 2020 and 2021, the number of buy recommendations increased significantly in all three subreddits, coinciding with the COVID-19 pandemic and the GameStop short squeeze. Overall, the WSB subreddit saw the largest increase in buy recommendations, while the investing and stocks subreddits had more stable and lower numbers.

The right panel of the table shows that the number of subscribers to each subreddit has also grown over time. WSB had the largest growth, especially from the beginning of 2021 to 2022 during the GameStop episode. This suggests that the popularity and importance of these subreddits, particularly WSB, have increased significantly in recent years.

### Portfolio formation

The Reddit ranking algorithm imposes a strong penalty on older submissions, which means that the time investors can interact with posts is relatively short, as they soon drop in rank and are replaced with new posts. Based on this fact, the main idea behind the construction of all portfolios is that at the end of each trading day, a hypothetical investor analyzes the community's submissions in the previous 24 h and buys the recommended stocks at the closing price. Each day the same amount is invested, and the size of the positions depends on the frequency of each ticker on that day. Formally, the return of stock j, which was featured in a submission on day d, can be measured as the change of the total return index from trading day d until day d + t using the following equation:

6 $${r}_{j, d}\left(t\right)=\frac{{TRI}_{j, d+t}-{TRI}_{j, d}}{{TRI}_{j, d}}$$

The portfolio weight x of that stock is then calculated by accumulating the number of relevant mentions of stock j over all relevant posts i in the 24 h prior to the close of the market and putting it in proportion to the sum of all mentions of all stocks in that same period:

7 $${x}_{j, d}\left(t\right)=\frac{{\sum }_{i}{Submission}_{i,j, d}}{{\sum }_{k}{\sum }_{i}{Submission}_{i,k, d}}$$

The return on the portfolio of stocks mentioned on trading day d is calculated by summing up the product of the stock return r and its portfolio weight x:

8 $${r}_{P, d}(t)={\sum }_{j}{r}_{j, d}\left(t\right)\cdot {x}_{j, d}\left(t\right)$$

We consider 1, 5, 21, 63 and 252 trading days as holding periods (t) which correspond roughly to one day, week, month, three months, and a year. This allows us to draw conclusions over both short and long holding periods.

### Risk-adjusted return

To analyze whether social media investors perform better than those who simply buy all available stocks or invest in an index fund, a suitable benchmark must be used. Investing in index funds is a simple and cost-effective alternative to investing in individual stocks for retail investors. Consequently, picking individual stocks based on social media posts, which involves screening cost, should result in higher returns to make it worthwhile. The US market returns from the data library of French (2022) are used to compare the realized returns.

However, it seems likely that the risk profile and factor exposure deviate from the market portfolio, making a simple comparison to the market return less meaningful. Thus, in addition, we use a Fama–French five-factor model (Fama & French, 2015) combined with the momentum factor (Jegadeesh & Titman, 1993) to control for factor exposure:

9 $${r}_{P}={r}_{F}+\alpha +{\beta }_{M}\left({r}_{M}-{r}_{F}\right)+{\beta }_{S}SMB+{\beta }_{V}HML+{\beta }_{P}RMW+{\beta }_{I}CMA+{\beta }_{Mom}MOM$$

In this equation ${r}_{P}$ is the return on the analyzed portfolio, ${r}_{F}$ is the risk-free return (1-month T-Bills are used) and ${r}_{M}$ is the return on the market portfolio. The coefficients indicate the factor exposure and will be determined by linear regressions: ${\beta }_{M}$ stands for the market risk, ${\beta }_{S}$ for the size factor, ${\beta }_{V}$ for the value factor, ${\beta }_{P}$ for the profitability factor, ${\beta }_{I}$ for the investment factor, ${\beta }_{Mom}$ for the momentum factor, and $\alpha$ is the factor-adjusted excess return. Fama and French’s (2015) definitions of SMB, HML, RMW and CMA are used as well as their daily data for these variables and MOM (French, 2022).

### CPT valuation

For CPT valuation, we use the approach described in Sects. 2.2 and 2.3 by substituting portfolio returns into the value (Eq. 2) and weighting (3 and 4) functions and calculating the sum of the product of the resulting values and probability weights (1). We use the market return as a reference point, i.e., we consider returns higher than the corresponding market return to be perceived as a gain, while lower returns are considered a loss. Therefore, the return used when calculating CPT values is the difference between the post-recommendation stock return and the market return.^7^ We then compute CPT values based on three widely used estimates. First, as in Barberis et al. (2016), we use the values of Tversky and Kahneman (1992): $\alpha =\beta =0.88;\,\gamma =0.61;\,\delta =0.69$ and $\lambda =2.25$. Second, we consider the average estimates of Rieger et al. (2017): $\alpha =0.46;\,\beta =0.58;\,\gamma =0.50;\,\delta =0.81$ and $\lambda =1.48$. Their sample features a large number of undergraduate students from economic-related fields from 53 different countries. Additionally, we use their estimates for the US: $\alpha =0.42;\,\beta =0.49;\,\gamma =0.44;\,\delta =0.71$ and $\uplambda =1.36$. These estimates are likely to be applicable to Reddit’s young and mostly male community (Boylston et al., 2021). Note that all the estimates rather underestimate the positive effects of the Reddit portfolio, as members of *wallstreetbets* are likely to have stronger preferences for lottery-like stocks than the average investor due to self-selection.

## Results

In this section, we first discuss the return distribution of Reddit and the market portfolio. Second, we calculate excess returns in a model that incorporates common risk factors. Third, we present the results of the valuation of these portfolios according to CPT.

### Return distribution

**Fig. 3:**
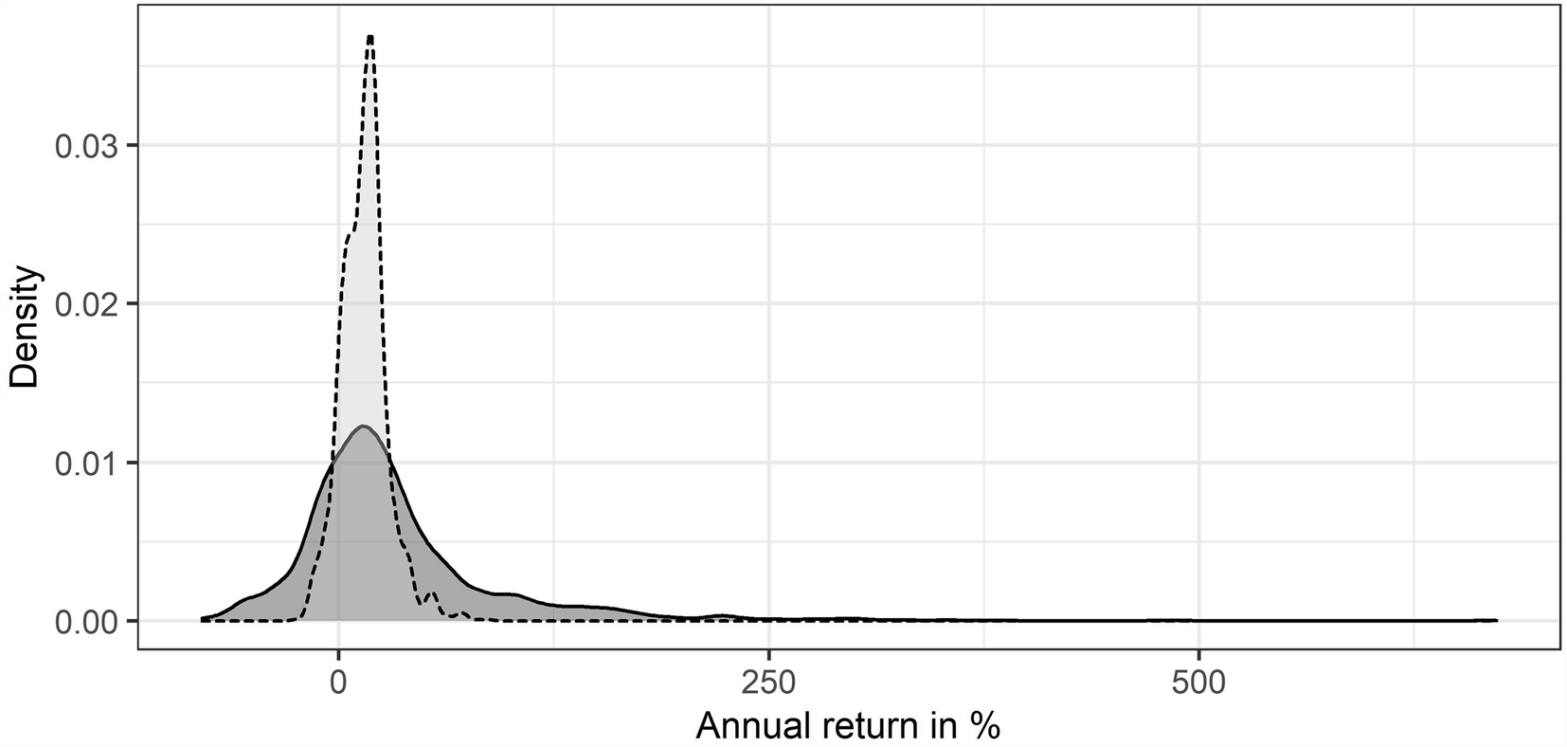
Density plot of the annual return of Reddit (in dark gray) and market portfolios (light gray and dashed line). The Reddit portfolio consists of recommended stocks from all three subreddits 24 h before market close, weighted by a number of recommendations. For each trading day, the return over the next 252 trading days is calculated

Figure 3 shows a plot of the density distribution of the returns of the Reddit portfolio based on all subreddits and the market portfolio over a one-year period. It is apparent that the returns of the market are more concentrated, and the Reddit portfolio has significantly larger tails.

A closer look at the descriptive statistics of the return distribution for different time horizons in Table 2 provides quantitative support for this observation.

**Table 2:** Descriptive statistics of the return distribution of the Reddit portfolio based on all subreddits and the market portfolio for different time horizons (measured in trading days)

Portfolio	Time Horizon	Mean	SD	Skewness	Median	Min	Max
Reddit	1	0.002	0.0513	6.5807	− 0.0002	− 0.518	1.0376
Market	1	0.0005	0.0111	− 0.6977	0.0007	− 0.1199	0.0935
Reddit	5	0.0097	0.1521	17.5438	0.0035	− 0.6544	4.7254
Market	5	0.0027	0.0229	− 0.9581	0.0042	− 0.1857	0.1682
Reddit	21	0.0334	0.217	10.319	0.0159	− 0.6663	4.8148
Market	21	0.0118	0.0438	− 1.4342	0.0162	− 0.3329	0.2284
Reddit	63	0.092	0.3219	4.8781	0.0459	− 0.7516	3.8751
Market	63	0.0362	0.069	− 0.4531	0.0412	− 0.306	0.4193
Reddit	252	0.3132	0.5791	2.4694	0.1928	− 0.7961	6.7294
Market	252	0.1474	0.1361	0.6796	0.1489	− 0.2527	0.8731

It is evident that the mean return of the Reddit portfolio is higher than the mean return of the market portfolio for all time horizons. However, the higher average return comes at the expense of a much higher risk, i.e., the Reddit portfolio’s standard deviations are four to six times higher than the ones of the market portfolio. Furthermore, the absolute values of the minimum returns, that is the maximum losses, are considerably higher. This suggests that investing in the Reddit portfolio is not attractive for investors that are risk averse. The Sharpe ratios presented in Table 2 provide quantitative support for this idea (Table 3).

**Table 3:** Sharpe ratios of the Reddit and market portfolio and the ratio of these two

Time horizon	Sharpe ratio reddit	Sharpe ratio market	Ratio of sharpe ratios
1	0.038	0.0392	0.9708
5	0.063	0.1128	0.5584
21	0.1519	0.259	0.5863
63	0.2811	0.5026	0.5593
252	0.5303	1.0272	0.5163

A ratio lower than one indicates that the Sharpe ratio of the Reddit portfolio is lower than the Sharpe ratio of the market portfolio

The Sharpe ratios of the Reddit portfolio, which represent the ratio of excess return and standard deviation as described by Sharpe (1994), are higher for the market portfolio for all time horizons. In particular, as shown in the last column, the portfolio only generates about half of the excess return per unit of absolute risk. This indicates that investing into the Reddit portfolio without additional diversification is not worthwhile for investors that use the mean–variance framework.

However, the Reddit portfolio has high maximum values, and its returns have a high positive skewness. In contrast, the market portfolio has a negative skewness for short time horizons. These high maximum returns and positive skewness might make investing in the Reddit portfolio attractive for investors with a preference for lottery-like stocks or bets.

Table 4 shows the expected returns in the 1st and 99th quantile of the distribution of the Reddit and market portfolio, respectively. The idea is that investors with CPT preferences overweight the tails of the distribution. Thus, the returns of “extreme” events are important for the valuation of a portfolio. Therefore, the last column contains the ratio of the expected return in the 99th quantile and the expected return in the 1st quantile, i.e., the ratio of the average of the highest percent and the lowest percent of returns. In the Reddit portfolio, the expected losses in the 1st quantile and the gains in the 99th quantile are higher than the corresponding values of the market portfolio, which reflects the higher risk. However, the expected gains in the tails are, on average, higher than the expected losses, which can be seen in the last column. For short-time horizons, the ratio of expected “extreme” gains to losses is higher for the Reddit portfolio than for the market portfolio. As these extreme events are overweighted, this result is a first indication that investors with CPT preferences might prefer to invest in the Reddit portfolio.

**Table 4:** Expected returns in the 1st and 99th quantile of the distribution of the Reddit and market portfolio

Portfolio	Time horizon	Expected return in the 1st quantile	Expected return in the 99th quantile	Ratio of the expected return in the 99th and 1st quantile
Reddit	1	− 0.1754	0.3071	1.7505
Market	1	− 0.0477	0.044	0.9239
Reddit	5	− 0.332	0.9276	2.7942
Market	5	− 0.1041	0.0803	0.7714
Reddit	21	− 0.4138	1.5034	3.6331
Market	21	− 0.2072	0.15	0.7238
Reddit	63	− 0.5187	2.2936	4.4217
Market	63	− 0.2236	0.2669	1.1934
Reddit	252	− 0.6592	3.2345	4.9064
Market	252	− 0.1721	0.6671	3.8768

### Risk-adjusted performance

Table 5 shows the results of the regression according to Fama and French (2015) with the addition of the momentum factor. Note that we only consider points of time for which data for all holding periods is available. In particular, posts in the last year of the observation period are not taken into account because no data on annual returns is available. Therefore, the number of considered stocks is the same for each time horizon, which makes the results for the different holding periods comparable. The daily excess returns (alphas) are initially positive up to a period of three months (corresponding to 63 trading days) but tend to decrease and become negative as soon as one year (252 trading days) is considered. Significant outperformance can be observed for the one- and three-month periods and significant underperformance for one year. These results indicate that the posts do not contain valuable information about mispriced companies, as the stocks recommended on Reddit (after controlling for risk factors) initially rise in the short-term but fall back below their starting level in the long-term. Therefore, we find no support for the idea that Redditors are able to use the wisdom of the crowd to identify undervalued opportunities. However, the pattern is in line with the opposing view, which states that the returns may be a result of “meme stocks”, meaning that the stocks are not worthwhile long-term investments for investors aiming to maximize alpha.

**Table 5:** Fama–French five-factor model including the momentum factor for the Reddit portfolio

Reddit portfolio	1	5	21	63	252
Daily alphas (in %)	0.1611	0.0289	0.0503***	0.0384***	− 0.0310***
${r}_{M}-{r}_{F}$	1.0989***	1.3199***	1.0482***	1.0588***	1.8199***
SMB	1.1706***	1.3886***	1.4959***	1.1769***	0.6396***
HML	− 0.5762^**^	− 0.8969***	− 0.2114	− 0.1457	− 1.1513***
RMW	− 0.4997	0.8113**	− 0.1235	0.5429***	0.7395***
CMA	2.4517***	3.2650***	0.8221***	0.3824	0.4199**
MOM	0.1281	0.2476*	− 0.0424	− 0.2713***	− 0.3644***
n	2298	2298	2298	2298	2298
R^2^	0.091	0.128	0.239	0.287	0.498

The first row indicates the time horizon measured as the number of trading days. Note that daily alphas are given in percent. The number of days on which buy recommendations were made and portfolios were formed is *n* = 2298

*p < 0.05, **p < 0.01,***p < 0.001

An analysis of the individual subreddit portfolios (Table 6) confirms that this pattern is also evident for WSB and, in less pronounced form, for *stocks* and *investing*. All three portfolios have significant negative annual excess returns. The alphas for other time periods are insignificant for *stocks* and *investing*. WSB has positive significant returns for holding periods of one month (21 trading days) and one quarter (63 trading days). These results are consistent with the notion that *wallstreetbets* focuses on short-term “bets” and its greater influence relative to the other subreddits may be driving stock prices up. However, following *investing* or *stocks* for long-term advice does not seem to be worthwhile as well.

**Table 6:** Daily excess returns in percent for the three subreddit portfolios

Subreddit portfolios	1	5	21	63	252
WSB	0.2126	0.0415	0.0613***	0.0601***	− 0.0357***
Stocks	0.0246	0.0161	0.0078	− 0.0076	− 0.0243***
Investing	0.0320	0.0215	− 0.0102	− 0.0071	− 0.0231**

The first row indicates the time horizon measured as the number of trading days. Note that daily alphas are given in percent

*p < 0.05, **p < 0.01, ***p < 0.001

### CPT valuations

In this subsection, we present the results of the CPT valuations of the Reddit portfolio post-recommendation returns using parameters according to Tversky and Kahneman (1992), and Rieger et al. (2017), respectively. Table 7 shows the results for the distribution of the returns for different holding periods. It is evident that the CPT valuations of the Reddit portfolio are positive for all time horizons when using the worldwide and US estimates of Rieger et al. (2017). The estimates of Tversky and Kahneman (1992) feature a higher loss aversion, meaning that high losses are weighted very strongly. The CPT values are positive for all holding periods except for one day. The negative sign for this very short time horizon may be explained by the chance of high losses that are weighted heavily. However, it is likely that investors that are members of subreddits like *wallstreetbets* do not have a strong loss aversion. Our results suggest that an investor with CPT preferences (and sufficiently low loss aversion) would prefer the return distribution of the Reddit portfolio over the return distribution of the market portfolio. For such an investor, investing in recommended stocks might thus be reasonable.

**Table 7:** CPT valuations of the Reddit portfolio for different holding periods

Time horizon	CPT Rieger	CPT Rieger US	CPT Tversky
1	0.0324	0.0197	− 0.0114
5	0.0596	0.0485	0.012
21	0.0761	0.0588	0.032
63	0.0987	0.0707	0.0603
252	0.1325	0.0905	0.124

We use the estimates of Rieger et al. (2017) for all countries ($\alpha =0.46;\,\beta =0.58;\,\gamma =0.50;\,\delta =0.81$; $\lambda =1.48$) and for the US ($\alpha =0.42;\,\beta =0.49; \,\gamma =0.44;\,\delta =0.71; \,\lambda =1.36$) and of Tversky and Kahneman (1992): $\alpha =\beta =0.88;\,\gamma =0.61;\,\delta =0.69$ and $\lambda =2.25$

Table 8 presents results divided by subreddit. The CPT valuations according to the estimates of Rieger et al. (2017) are positive for all subreddits and time horizons. This indicates that the positive skewness can compensate for the disadvantages in mean and variance. When using the estimates of Tversky and Kahneman (1992), the results for the WSB subreddit are consistent with the results for all subreddits in Table 7. However, the other subreddits have negative CPT valuations for all time horizons except for one year. The result that WSB has consistently the highest CPT values may be explained by the subreddit’s goal. Agrawal et al. (2022) show that WSB mainly discusses high-risk strategies. Furthermore, the name *wallstreetbets* suggests that the members of the subreddit try to identify interesting betting targets. These targets are likely to be appealing to investors with preferences for lottery-like stocks as in CPT. The fact that the subreddits *stocks* and *investing* only have positive CPT values for long time horizons using the estimates of Tversky and Kahneman (1992) fits with the notion that these subreddits focus more on long-term investing and its members are likely to place less emphasis on short-term returns. For investors with preferences such as in CPT and long-time horizons, following the long-term stock recommendations is appealing because the corresponding CPT values are positive.

**Table 8:** CPT valuations divided by subreddit for different holding periods

Subreddit	Time horizon	CPT Rieger	CPT Rieger US	CPT Tversky
WSB	1	0.0326	0.0178	− 0.0158
WSB	5	0.0629	0.0505	0.0152
WSB	21	0.0825	0.0638	0.0459
WSB	63	0.1211	0.0912	0.1017
WSB	252	0.1722	0.1248	0.1998
Stocks	1	0.0203	0.0058	− 0.0189
Stocks	5	0.0205	0.0005	− 0.0295
Stocks	21	0.0366	0.0197	− 0.0167
Stocks	63	0.0241	0.0018	− 0.037
Stocks	252	0.0588	0.0286	0.0582
Investing	1	0.0207	0.0057	− 0.02
Investing	5	0.0319	0.0144	− 0.0191
Investing	21	0.0052	− 0.0202	− 0.0561
Investing	63	0.0256	0.0028	− 0.0413
Investing	252	0.1006	0.0758	0.1523

We use the estimates of Rieger et al. (2017) for all countries ($\alpha =0.46;\,\beta =0.58;\,\gamma =0.50;\,\delta =0.81$; $\lambda =1.48$) and for the US ($\alpha =0.42;\,\beta =0.49;\,\gamma =0.44;\,\delta =0.71$; $\lambda =1.36$) and of Tversky and Kahneman (1992): $\alpha =\beta =0.88; \,\gamma =0.61;\, \delta =0.69$ and $\lambda =2.25$

To sum up, our results indicate that investing in the Reddit portfolio would be attractive for investors with preferences as in CPT, especially when using the estimates of Rieger et al. (2017), which are more likely to be representative of Redditors.

## Discussion

In this section, we discuss the implications and limitations of our study.

As the Sharpe ratios of the Reddit portfolio are smaller than the ones of the market, following the stock recommendations without further diversification is not worthwhile for an investor using the mean–variance framework. When considering risk factors according to Fama and French (2015) with the addition of the momentum factor, the Reddit portfolio shows positive alphas in the short-term, while alphas are significantly negative for longer time horizons for all three subreddits. This indicates that the Reddit community does not recommend stocks that are underpriced from a mean–variance point of view, and, therefore, yield long-term outperformance. Instead, the observed pattern, especially on WSB, is consistent with the idea of “meme stocks”, i.e., that prices of recommended stocks get inflated by the community until it loses interest, and prices return to the stocks’ fundamental values. This suggests that Reddit posts might cause herd behavior and do not contain valuable information about long-term success.

However, if Redditors value returns in terms of CPT and not the mean–variance framework, insignificant or negative alphas do not necessarily imply that the stocks are not appealing to these investors. In fact, we show that investing in the Reddit portfolio is reasonable for CPT investors with parameters according to Tversky and Kahneman (1992) or Rieger et al. (2017), respectively. WSB in particular has favorable CPT values, while the other two subreddits only have favorable CPT values for (relatively) long time horizons. However, *investing* and *stocks* mainly discuss long-term investments, which is why following the long-term recommendations may be reasonable for a CPT investor.

Overall, our findings suggest that Reddit posts contain information. However, it is not about Sharpe ratios or alphas but about stocks that have desirable characteristics from a CPT point of view. In other words, our findings indicate that WSB does exactly what its name suggests: it discusses stocks that are attractive to investors with preferences for betting and lottery-like stocks. These discussions lead to social interactions, and attention on lottery-like stocks, which in turn may amplify the lottery anomaly. Furthermore, the pattern of alphas is consistent with attention-based trading. Therefore, our results to some extent support the conclusion of Bali et al. (2021) that attention and social interactions contribute to investors’ attraction to lottery-like stocks. Furthermore, our results are in line with Hu and Yan (2022) who find that social media attention drives retail investor trading activity, and that social media attention is informative. However, we do not directly examine which stock characteristics (such as momentum or skewness of the recommended stock’s historical returns) capture the attention of Redditors and motivate them to post about these stocks. This is a promising area for future research.

Our results are not only relevant for retail investors using social media but also for more sophisticated investors, such as hedge funds. Specifically, they could try to take advantage of the pattern of excess returns by shorting stocks that were recommended more than three months ago and buying recently recommended stocks. Another promising application of social media data in the future would be to use it to predict which stocks will become meme stocks soon. This is especially relevant for short sellers of these stocks to avoid being on the losing side of a short squeeze. Investors could also buy “hot” stocks in the hope of capturing their short-term alphas and being able to sell them in time when social media attention and consequently returns decrease.

Our study contributes to the literature on financial advice on social media, and on Reddit in particular, by presenting a behavioral explanation of why the recommendations are followed, although no significant positive alphas can be generated. Moreover, our findings indicate that Reddit posts may contain valuable information about, e.g., the skewness of stock returns. Extracting, and using these may be a promising task for future research. Furthermore, the results highlight the relevance of CPT in finance, as it shows that CPT is consistent with the behavior of millions of Redditors.

Finally, it must be noted that our study has several limitations. First, although our data set is large and covers a long period, small gaps and inaccuracies in both Pushshift's Reddit data and Eikon's financial data exist. Second, although we achieve a higher precision and recall than Chacon et al. (2022) using sentiment analysis, the Reddit buy portfolios we construct still contain some sell recommendations (around 3.5%). However, better identification of buy recommendations would likely strengthen our results, as these sell recommendations are likely associated with undesirable characteristics for investors. Third, no taxes or trading fees (including bid-ask spreads) have been taken into account. These could have a significant impact on returns, especially for short holding periods. However, the general outcome is unlikely to change as the benchmarks are calculated for the same periods and would also incur taxes and fees. Fourth, we do not control for events like the disclosure of quarterly reports that may be related to the Reddit posts and explain the resulting stock return distributions. However, we do not claim that the relationship between post and return distributions is causal. In fact, when considering whether it is reasonable to follow the stock recommendations on Reddit, it does not matter whether the information is available exclusively on Reddit but rather that the posts are associated with favorable characteristics of the return distribution. Furthermore, the costs of reading quarterly reports are likely to be much higher than the costs of reading Reddit posts. Therefore, even when Reddit posts only contain information from these reports, they still can add value by reducing the costs of acquiring this information.

## Summary

This study investigates stock recommendations on the three largest financial subreddits on Reddit, i.e., *wallstreetbets*, *stocks*, and *investing*. We consider data from 2008 to August 2022 and use a simple investment strategy: Recommended stocks are bought and weighted by the number of posts. The resulting portfolios are assessed based on different time horizons and performance metrics. We find that the Sharpe ratios are unfavorable, which implies that following stock recommendations without further diversification is not worthwhile in the mean–variance framework. The alphas based on a five-factor model with the addition of the momentum factor are positive in the short run but become negative for longer time horizons. This suggests that the return distribution of recommended stocks is more consistent with the idea of “meme stocks”, meaning that prices get inflated in the short run, and return to the fundamental value once interest is lost. Thus, Reddit posts do not seem to be associated with favorable characteristics in terms of the mean–variance framework.

However, we consider valuations according to cumulative prospect theory and show that the CPT valuations of the Reddit portfolio are positive. This indicates that following the stock recommendations may be reasonable for an investor with preferences as in CPT. This implies that Reddit posts may contain information on stock characteristics such as the skewness of stock returns. Extracting and using these is a promising task for future research. Overall, our study contributes to the literature by providing a behavioral explanation for the behavior of Redditors, which may be a foundation for future research.

## Data Availability

The data that support the findings of this study are available from the corresponding author, MW, upon reasonable request.

## Footnotes

1 See, e. g., Stacey (2021) in the Financial Times and Mackintosh (2021) in the Wall Street Journal.

2 The backtests examine whether following Reddit recommendations in the past would have led to outcomes that investors would have regretted or been satisfied with, given their preferences. Thus, the backtests indicate whether following Reddit posts would have been a good (reasonable) decision. Furthermore, when investors take the past realized returns as a reference (and assume that the return distribution will be similar in the future), favorable backtests would mean that investors with the respective preferences should use Reddit recommendations when investing today.

3 From WSB’s first post: “I know that /r/investing is a great place for congregating with fellow market gamblers—but it's not exactly the right place to be. So rather than flooding it with gambling posts and epic win/loss screen shots I figured I'd make another sub for this specifically for speculating and such. We'll see if it works. Subscribe if you're interested […]”.

4 The GradyAugmented word list from the qdapDictionaries package was used. See Rinker (2018).

5 These include, among others, the “rocket”, “gem stone”, and “crescent moon”, which are the most frequently used emojis in our dataset.

6 Words that indicate a buy signal are buy, bought, moon, hold, call, bull, like, moon and yolo. Words that indicate a sell signal are sell, bear, liquidate, sold, put.

7 Note that the results in Sect. 4.3 are independent of whether excess returns or nominal returns are used to calculate the CPT values. When a reference return of zero is used to calculate the CPT values of the Reddit portfolio and the corresponding market portfolio, the CPT value of the Reddit portfolio is higher, indicating the same decision as when the market return is used as the reference point.

## References

[CR2] Agrawal, P., Buz, T, & de Melo, G. (2022). WallStreetBets beyond GameStop, YOLOs, and the moon: the unique traits of reddit’s finance communities. twenty-eighth Americas conference on information systems. http://gerard.demelo.org/papers/wallstreetbets-social.pdf.

[CR3] Amaya D, Christoffersen P, Jacobs K, Vasquez A (2015). Does realized skewness predict the cross-section of equity returns?. Journal of Financial Economics.

[CR4] An L, Wang H, Wang J, Jianfeng Y (2020). Lottery-related anomalies: the role of reference-dependent preferences. Management Science.

[CR5] Antweiler W, Frank MZ (2004). Is all that talk just noise? The information content of internet stock message boards. The Journal of Finance.

[CR6] Bali TG, Cakici N, Whitelaw RF (2011). Maxing out: stocks as lotteries and the cross-section of expected returns. Journal of Financial Economics.

[CR7] Bali T, Hirshleifer D, Peng L, Tang Y (2021). Attention, social interaction, and investor attraction to lottery stocks. National Bureau of Economic Research.

[CR8] Barberis N, Huang M (2008). Stocks as lotteries: the implications of probability weighting for security prices. American Economic Review.

[CR9] Barberis N, Jin L, Wang B (2021). Prospect theory and stock market anomalies. The Journal of Finance.

[CR10] Barberis N, Mukherjee A, Wang B (2016). Prospect theory and stock returns: an empirical test. The Review of Financial Studies.

[CR11] Bartov E, Faurel L, Mohanram PS (2018). Can twitter help predict firm-level earnings and stock returns?. The Accounting Review.

[CR12] Baumgartner, J., Zannettou, S., Keegan, B., Squire, M., & Blackburn, J. (2020). The pushshift reddit dataset. *Proceedings of the International AAAI Conference on Web and Social Media* (14).

[CR13] Betzer A, Harries J. P. (2022). “How online discussion board activity affects stock trading: the case of GameStop. Financial Markets and Portfolio Management.

[CR14] Boylston, C., Palacios, B., Tassev, P, & Bruckman, A. (2021). Wallstreetbets: positions or ban. https://arxiv.org/pdf/2101.12110.

[CR15] Bradley, D., Hanousek Jr, J., Jame, R., & Xiao, Z. (2021). Place your bets? The market consequences of investment advice on reddit’s Wallstreetbets. SSRN 3806065.

[CR16] Buz, T., & de Melo, G. (2021). Should you take investment advice from wallstreetbets? A data-driven approach. arXiv preprint arXiv:2105.02728.

[CR17] Chacon RG, Morillon TG, Wang R (2022). Will the reddit rebellion take you to the moon? Evidence from WallStreetBets. Financial Markets and Portfolio Management.

[CR18] Chen H, De Prabuddha Y, Hwang B-H (2014). Wisdom of crowds: the value of stock opinions transmitted through social media. The Review of Financial Studies.

[CR19] Chohan, U. W. (2021). Too big to fail, too small to win: the counter-hegemony of wallstreetbets. SSRN 3849770.

[CR20] Corbet, S., Hou, G., Hu, S., & Oxley, L. (2021). We reddit in a forum: the influence of messaging boards on firm stability. SSRN 3776445.

[CR21] Costola, M., Iacopini, M., & Santagiustina, C. R. (2021). On the ”Mementum” of meme stocks. arXiv preprint arXiv:2106.03691.

[CR22] Eraker B, Ready M (2015). Do investors overpay for stocks with lottery-like payoffs? An examination of the returns of OTC stocks. Journal of Financial Economics.

[CR23] Fama EF, French KR (2015). A five-factor asset pricing model. Journal of Financial Economics.

[CR24] Farrell M, Green TC, Jame R, Markov S (2022). The democratization of investment research and the informativeness of retail investor trading. Journal of Financial Economics.

[CR25] Fong WM, Toh B (2014). Investor sentiment and the MAX effect. Journal of Banking & Finance.

[CR26] French, K. R. (2022). Data Library. https://mba.tuck.dartmouth.edu/pages/faculty/ken.french/data_library.html. Accessed 06 Sept 2022.

[CR27] Giannini R, Irvine P, Shu T (2018). Nonlocal disadvantage: an examination of social media sentiment. The Review of Asset Pricing Studies.

[CR28] Hasso T, Müller D, Pelster M, Warkulat S (2021). Who participated in the Gamestop frenzy? Evidence from brokerage accounts. Finance Research Letters.

[CR29] He XD, Zhou XY (2011). Portfolio choice under cumulative prospect theory: an analytical treatment. Management Science.

[CR30] Hutto C, Gilbert E (2014). Vader: a parsimonious rule-based model for sentiment analysis of social media text. Proceedings of the International AAAI Conference on Web and Social Media.

[CR31] Jegadeesh N, Titman S (1993). Returns to buying winners and selling losers: implications for stock market efficiency. The Journal of Finance.

[CR32] Jia W, Redigolo G, Shu S, Zhao J (2020). Can social media distort price discovery? Evidence from merger rumors. Journal of Accounting and Economics.

[CR33] Kumar A (2009). Who gambles in the stock market?. The Journal of Finance.

[CR34] Loughran T, McDonald B (2011). When is a liability not a liability? Textual analysis, dictionaries, and 10-Ks. The Journal of Finance.

[CR35] Lyócsa Š., Baumöhl E, Výrost T (2022). YOLO trading: riding with the herd during the gamestop episode. Finance Research Letters.

[CR36] Mackay, C. (1841). Memoirs of extraordinary popular delusions. London. https://books.google.de/books?id=ufoLAAAAYAAJ.

[CR37] Mackintosh, J. (2021). GameStop Is a Bubble in Its Purest Form. https://www.wsj.com/articles/gamestop-is-a-bubble-in-its-purest-form-11611756239. Accessed 18 Aug 2021.

[CR38] Mitton T, Vorkink K (2007). Equilibrium underdiversification and the preference for skewness. The Review of Financial Studies.

[CR39] Reddit. (2021). R/wallstreetbets - WSB AMA - LIVE NOW. https://www.reddit.com/r/wallstreetbets/comments/l7yc12/wsb_ama_live_now/. Accessed 12 Oct 2022.

[CR1] Reddit. (2022). Wallstreetbets: Rules. https://www.reddit.com/r/wallstreetbets/about/rules. Accessed 12 Oct 2022.

[CR40] Rieger MO, Wang M, Hens T (2017). Estimating cumulative prospect theory parameters from an international survey. Theory and Decision.

[CR41] Rinker, T. (2018). QdapDictionaries: dictionaries and word lists for the ‘Qdap’ Package. https://cran.r-project.org/web/packages/qdapDictionaries/qdapDictionaries.pdf. Accessed 26 Aug 2021.

[CR42] Sasaki Y (2007). The truth of the f-measure. Teach Tutor Mater.

[CR43] Semenova, V., & Winkler, J. (2021). Reddit’s Self-Organised Bull Runs: Social Contagion and Asset Prices. arXiv preprint arXiv:2104.01847.

[CR44] Sharpe WF (1994). The sharpe ratio. Journal of Portfolio Management.

[CR45] Singer, P., Flöck, F., Meinhart, C., Zeitfogel, E., & Strohmaier, M. (2014). Evolution of Reddit: From the Front Page of the Internet to a Self-Referential Community? *Proceedings of the 23rd International Conference on World Wide Web*.

[CR46] Stacey, K. (2021). GameStop Mania: Why Reddit Traders Are Unlikely to Face Prosecution. https://www.ft.com/content/8caa3c75-944a-468e-8a68-9deeec8b67d8. Accessed 18 Aug 2021.

[CR47] Surowiecki J (2004). The wisdom of crowds: why the many are smarter than the few and how collective wisdom shapes business, economies, societies, and nations.

[CR48] Tang VW (2018). Wisdom of crowds: cross-sectional variation in the informativeness of third-party-generated product information on twitter. Journal of Accounting Research.

[CR49] Tversky A, Kahneman D (1992). Advances in prospect theory: cumulative representation of uncertainty. Journal of Risk and Uncertainty.

[CR50] Walther M, Münster M (2021). Conditional risk premiums and the value function of prospect theory. Journal of Behavioral Finance.

[CR51] Wang H, Yan J, Jianfeng Y (2017). Reference-dependent preferences and the risk–return trade-off. Journal of Financial Economics.

[CR52] Weiting H, Yan R (2022). Social media attention, stock returns and retail trades.

[CR53] Welch I (2022). The wisdom of the robinhood crowd. The Journal of Finance.

